# The transducer-like protein Tlp12 of *Campylobacter jejuni* is involved in glutamate and pyruvate chemotaxis

**DOI:** 10.1186/s12866-018-1254-0

**Published:** 2018-09-10

**Authors:** Anastasia-Lisa Lübke, Sabrina Minatelli, Thomas Riedel, Raimond Lugert, Isabel Schober, Cathrin Spröer, Jörg Overmann, Uwe Groß, Andreas E. Zautner, Wolfgang Bohne

**Affiliations:** 1 Institute for Medical Microbiology, University Medical Center, Kreuzbergring 57, 37075 Göttingen, Germany; 2 Leibniz Institute DSMZ-German Collection of Microorganisms and Cell Cultures, Braunschweig, Germany; 3 German Center for Infection Research (DZIF), Partner Site Hannover–Braunschweig, Braunschweig, Germany

**Keywords:** *Campylobacter jejuni*, Chemoreceptor, Transducer-like protein, Chemotaxis, Glutamate, Pyruvate, Tlp12

## Abstract

**Background:**

*Campylobacter jejuni* is one of the most common bacterial causes of food-borne enteritis worldwide. Chemotaxis in *C. jejuni* is known to be critical for the successful colonization of the host and key for the adaptation of the microbial species to different host environments. In *C. jejuni,* chemotaxis is regulated by a complex interplay of 13 or even more different chemoreceptors, also known as transducer-like proteins (Tlps). Recently, a novel chemoreceptor gene, *tlp12*, was described and found to be present in 29.5% of the investigated *C. jejuni* strains.

**Results:**

In this study, we present a functional analysis of Tlp12 with the aid of a *tlp12* knockout mutant of the *C. jejuni* strain A17. Substrate specificity was investigated by capillary chemotaxis assays and revealed that Tlp12 plays an important role in chemotaxis towards glutamate and pyruvate. Moreover, the Δ*tlp12* mutant shows increased swarming motility in soft agar assays, an enhanced invasion rate into Caco-2 cells and an increased autoagglutination rate. The growth rate was slightly reduced in the Δ*tlp12* mutant. The identified phenotypes were in partial restored by complementation with the wild type gene. Tlp12-harboring *C. jejuni* strains display a strong association with chicken, whose excreta are known to contain high glutamate levels.

**Conclusions:**

TLP12 is a chemoreceptor for glutamate and pyruvate recognition. Deletion of *tlp12* has an influence on distinct physiological features, such as growth rate, swarming motility, autoagglutination and invasiveness.

**Electronic supplementary material:**

The online version of this article (10.1186/s12866-018-1254-0) contains supplementary material, which is available to authorized users.

## Background

Chemotaxis is a critical mechanism for the successful colonization of host animals and ecological niches by *Campylobacter* spp. [1, 2]. Their movement towards favorable chemical stimuli such as amino acid carbon sources and away from unfavorable stimuli, e.g. bile acids, drives their movement to niches of optimal growth conditions. Chemotaxis receptors, also referred to as methyl-accepting chemotaxis proteins or transducer-like proteins (Tlps), activate flagellar motility by detecting various chemical stimuli in the environment and triggering a signal transduction cascade, which modulates the flagellar motor [3].

Chemotaxis in *C. jejuni* is mediated by – according to current knowledge – 13 transducer-like proteins (Tlps) and two aerotaxis proteins (Aer) [4–6]. These chemoreceptors have been grouped into three subtypes, namely A, B and C [5]. Group-A Tlps are integral membrane proteins consisting of two transmembrane domains, an *N*-terminal, periplasmic, sensory domain that binds chemotaxis ligands and a cytoplasmic signaling domain. Tlps in this group are Tlp1, Tlp2, Tlp3, Tlp4, Tlp7, Tlp10, Tlp11, Tl12, and Tlp13. The presence of a periplasmic ligand-binding domain allows the group A Tlps to sense ligands external to the cell. Tlp9 (CetA) is the only group B Tlp. It consists of an *N*-terminal transmembrane domain anchoring the receptor in the inner membrane of the bacterium and a *C*-terminal cytoplasmic signaling region. It interacts with two cytoplasmic ligand proteins to sense aerotactic signals (Aer1 and Aer2) [6]. The group C Tlps include Tlp5, Tlp6 and Tlp8 and are cytoplasmic proteins that detect cytosolic signals [5]. All Tlps are thought to form homodimers and arrange in groups of three [5].

Even though the identification of specific chemoeffectors for the individual Tlps is often complicated due to the frequent compensation of the function of one chemoreceptor for another [7], the specificities of a few TLPs have successfully been identified. Aspartate was found to be the chemoattractant of Tlp1, Tlp3, Tlp6 and Tlp10 [8–10]. Tlp6 and Tlp10 have also been found to be chemoreceptors for glutamine and/or glutamate and TCA cycle intermediates [8]. Formic acid has been identified as chemoattractant of Tlp7 [11, 12]. Tlp3 has been identified as a chemoreceptor for multiple chemoeffectors, therefore named “*Campylobacter* chemoreceptor for multiple ligands” (CcmL) [10]. Bile acids are usually chemorepellents and are sensed by Tlp3 and Tlp4 [13].

A study by Mund et al. [14] revealed that *tlp1, tlp2, tlp3, tlp6, tlp8, tlp9* and *tlp10* were all conserved, whereas the *tlp4, tlp5, tlp7*, *tlp11*, *tlp12*, and *tlp13* genes were significantly variable. The *tlp4* gene (*cj0262c*) in the *C. jejuni* strain NCTC 11168 is flanked by a gene encoding a hypothetical protein (*cj0261c*) and *zupT* (*cj0263c*). Several other *C. jejuni* strains possess at the same position a *tlp* gene that displays a significantly different sensory domain sequence compared to the *C. jejuni* NCTC 11168 *tlp4* gene. In the *C. jejuni* R14 strain for example, 173 bp of the 5′ end and 1136 bp of the 3′ end of this *tlp* (H730_01610*)* were highly similar to the *tlp4* gene in NCTC 11168, however, the 682 bp sequence in between shared no significant sequence identity [14]. Thus, the *C. jejuni* R14 gene (H730_01610)*,* was hypothesized to have a different function to *tlp4* and homologs of this gene were named *tlp12* [14]. The *tlp12* gene is not ubiquitous among *C. jejuni* strains. From 292 analyzed *C. jejuni* isolates, 29.5% were found to possess the *tlp12* gene [14].

In this study, we performed a functional analysis of Tlp12 with the aid of a *Δtlp12* mutant generated in a strain that naturally lacks *tlp4*, *tlp11*, and *tlp13*, as demonstrated by single-molecule real-time (SMRT) genome sequencing. We systematically investigated the chemotactic behavior of this mutant to a variety of compounds that were previously shown to be involved in *C. jejuni* chemotaxis and could reveal that pyruvate and glutamate are chemoattractors for Tlp12. Moreover, secondary roles of Tlp12 for motility, autoagglutination and biofilm formation were elucidated.

## Results

For functional *tlp12* analysis, we chose the *C. jejuni* human stool isolate A17, which contains *tlp12*, but lacks *tlp4*, *tlp11*, and *tlp13*. *Tlp4* was previously shown to share a smaller *N*-terminal and a larger *C*-terminal region with *tlp12* (Mund et al. 2016 [14]) and attempts to delete *tlp12* in a *tlp4*-positive *C. jejuni* strain might lead to incorrect integration of the targeting construct. The presence of *tlp12* and absence of *tlp4* in the A17 isolate was initially detected by PCR [14] and subsequently confirmed by analysis of its complete genome sequence. The genome of the *C. jejuni* strain A17 consists of a circular chromosome of 1,683,497 bp and an extrachromosomal element of 134,322 bp. The chromosome contains 1660 predicted coding sequences (CDS) with a coding density of 0.986 genes per kb and an average gene size of 928 bp, 9 rRNAs and 44 tRNAs. The G + C content is 30.42%. The extrachromosomal element encodes 152 CDS with a coding density of 1.13 genes per kb and an average gene size of 772 bp.

**Fig. 1:**
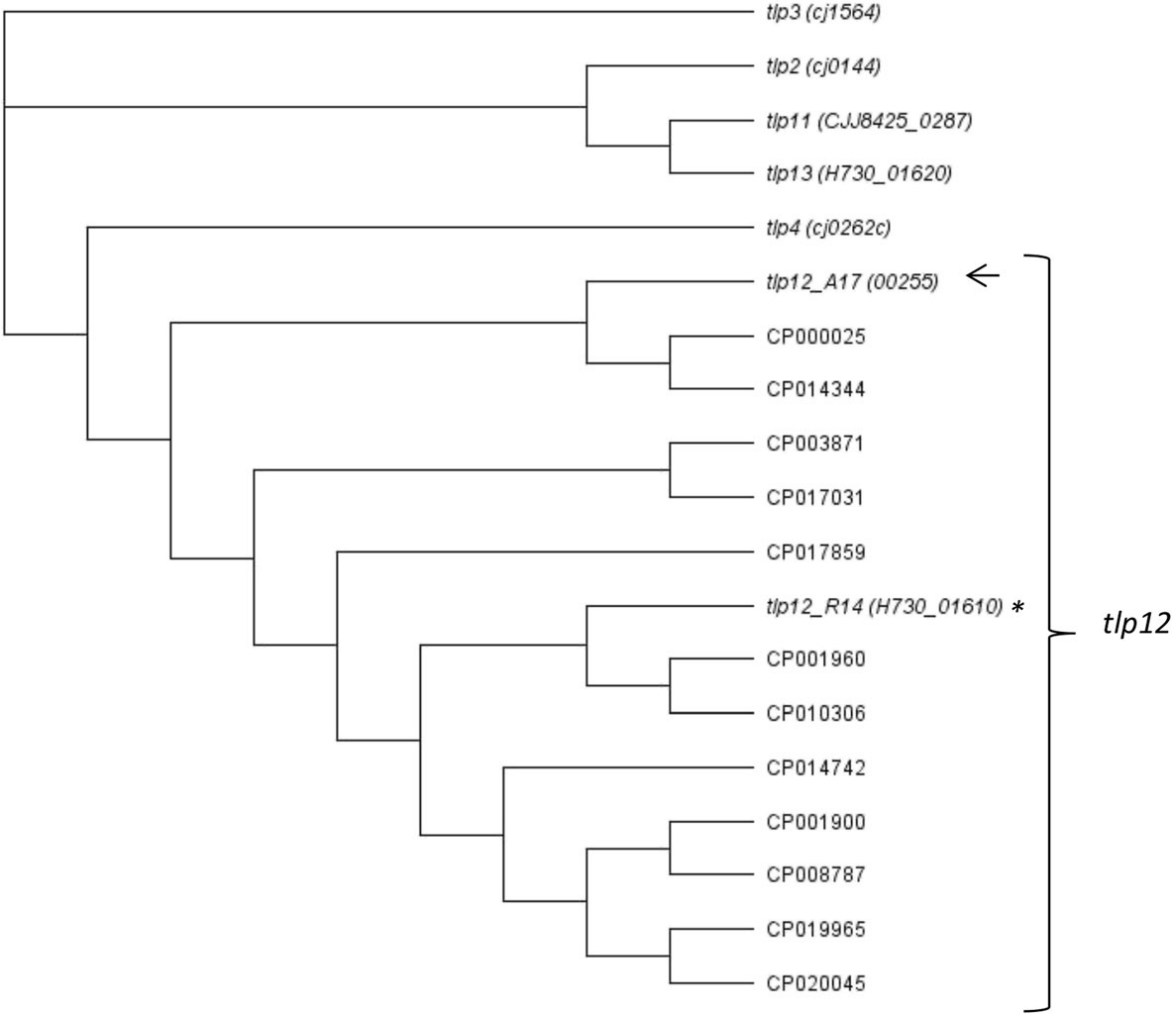
Neighbor-joining tree. The top thirteen hits of a nucleotide BLAST search using the coding sequence of *tlp12* of strain A17 as a query were aligned with the *tlp12* sequence of strain A17 together with the reference sequences for *tlp2*, *tlp3*, *tlp4*, *tlp11* and *tlp13*. Global alignment with free end gaps was used. A neighbor-joining tree was constructed using the Geneious 10.0.9 software. The *tlp12* CDS of strainA17 is indicated by an arrow, the *tlp12* CDS of strain R14 is indicated by an asterisk

The coding region of the A17 *tlp12* gene predicts a protein of 662 amino acids with a molecular weight of 73.2 kDa. The putative domain structure and an alignment with Tlp12 of strain R14 (H730_01610) and Tlp4 of strain NCTC 11168 (Cj0262c) are shown in Additional file 1. A neighbor-joining tree was constructed with the nucleotide sequences of the top thirteen results of a BLAST using the A17 *tlp12* CDS as a query. For comparison, reference sequences of the five *tlp* genes that are most similar to *tlp12* of strain A17 (*tlp2, tlp3, tlp4, tlp11 and tlp13* of NCTC 11168) were included in the neighbor-joining tree. The neighbor-joining tree indicates that *tlp12_A17* of strain A17 is most closely related to *tlp4* of strain NCTC 11168, among the *tlp* gene reference genes (Fig. 1). Furthermore, the tree reveals a considerable amount of variability among the *tlp12* genes obtained from the strain A17 *tlp12* BLAST search. Sub-clades of the *tlp12* gene can be seen among the fourteen *C. jejuni* isolates, which include R14, the strain in which the *tlp12* gene was originally identified. A17 *tlp12* and R14 *tlp12* are seen to be located within different sub-clades. Whether sub-clades are associated with functional differences between the distinct *tlp12* alleles remains to be investigated.

**Table 1:** Chemotactic response

Compound	WT RCR	*Δtlp12* RCR
Aspartate	0.97 ± 0.01	0.28 ± 0.03
Cysteine	0.06 ± 0.03	0.04 ± 0.01
Fumarate	0.68 ± 0.27	0.70 ± 0.05
Glutamate	18.10 ± 5.60	2.33 ± 1.38
Glutamine	1.80 ± 0.01	2.80 ± 0.16
Lactate	3.50 ± 0.45	6.30 ± 0.26
Pyruvate	7.90 ± 0.18	1.83 ± 0.12
Serine	2.01 ± 0.31	1.47 ± 0.01
Succinate	0.50 ± 0.17	1.53 ± 0.14

### Glutamate and pyruvate are chemoattractors for Tlp12

A syringe-capillary assay was used to determine the chemotactic response of the wild type A17 isolate towards compounds that have been shown previously to act as chemoattractors in *C. jejuni*. The collection of tested compounds included the five amino acids serine, glutamine, aspartate, cysteine and glutamate and the four intermediates of the central carbon metabolism fumarate, succinate, lactate and pyruvate. All compounds were used at concentrations of 100 mM. A compound was considered as a chemoattractant when relative chemotactic response (RCR)-values were > 1.8, which was the case for lactate, serine, glutamine, glutamate and pyruvate (Table 1).

**Fig. 2:**
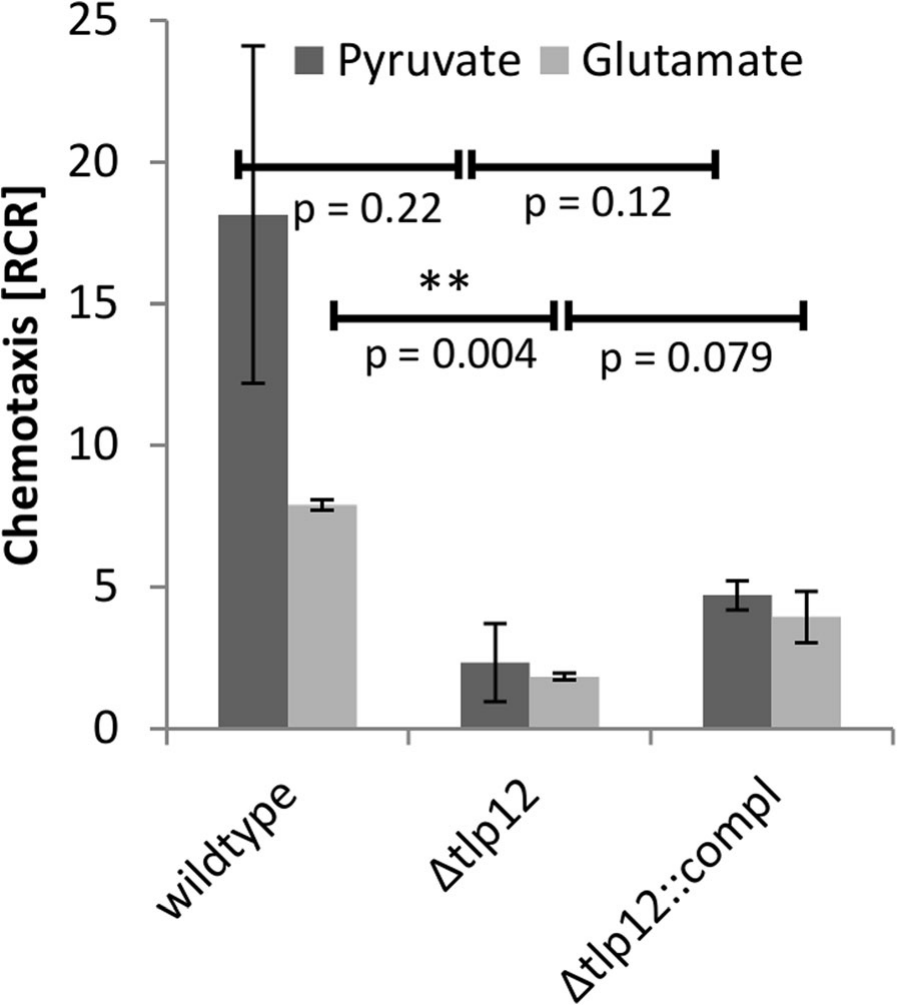
Chemotaxis assay. Syringe-capillary assays were performed with 100 mM glutamate and 100 mM pyruvate as chemoattractants. Error bars show the standard deviation from three biological replicates, each performed in technical duplicates or triplicates for glutamate chemotaxis and a representative experiment, performed in technical triplicates for pyruvate chemotaxis. The *tlp12* mutant shows strongly reduced chemotaxis towards glutamate and pyruvate. **p < 0.005

To test the contribution of Tlp12 to chemotaxis towards these compounds, we generated a *Δtlp12* mutant of strain A17 by integration of a kanamycin cassette via double homologous recombination into the *tlp12* locus. Complementation was performed by a plasmid-based, double-recombinational insertion of the *tlp12*-coding sequence, linked to a chloramphenicol resistance cassette (CamR), into one of the three rRNA loci of the A17 *Δtlp12* mutant (Additional file 2). Subsequently, syringe-capillary assays were performed in order to compare the chemotaxis of the mutant with the parental strain A17. The *Δtlp12* mutant showed a significant (*p* = 0.0044) reduction of chemotaxis to glutamate (13% of wild type level), which was partially restored in the complemented mutant (Fig. 2), suggesting that Tlp12 seems to play a major role in glutamate chemotaxis. Pyruvate chemotaxis was reduced in the *Δtlp12* mutant to 23% of wild type level, however, *p*-values for pyruvate chemotaxis failed to show statistical significance (*p* > 0.05).

### The *Δtlp12* mutant shows increased swarming motility, invasion and autoagglutination, but a decreased growth rate

**Fig. 3:**
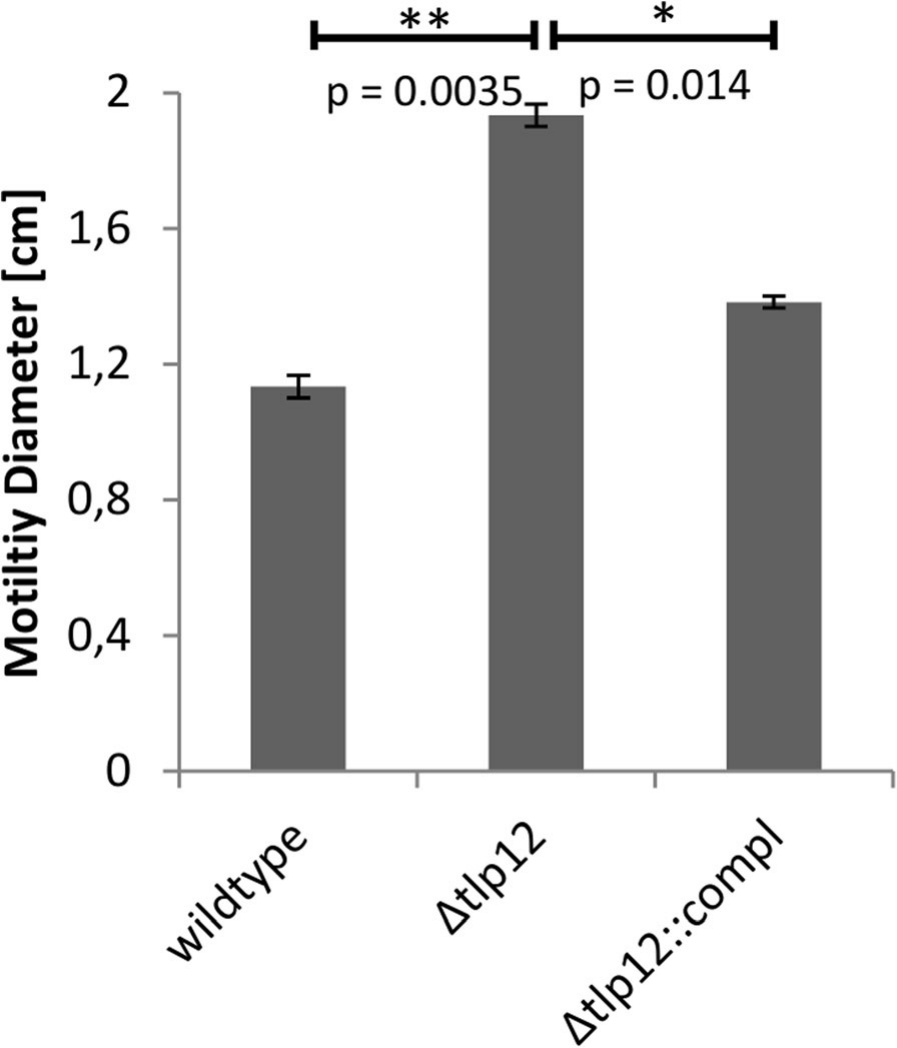
Soft agar swarming motility assay. The diameter of the migration zone was determined in soft agar. Motility experiments were performed in technical and biological triplicates. Error bars show the standard deviation of a representative experiment (*n* = 3). *p < 0.05; **p < 0.005

**Fig. 4:**
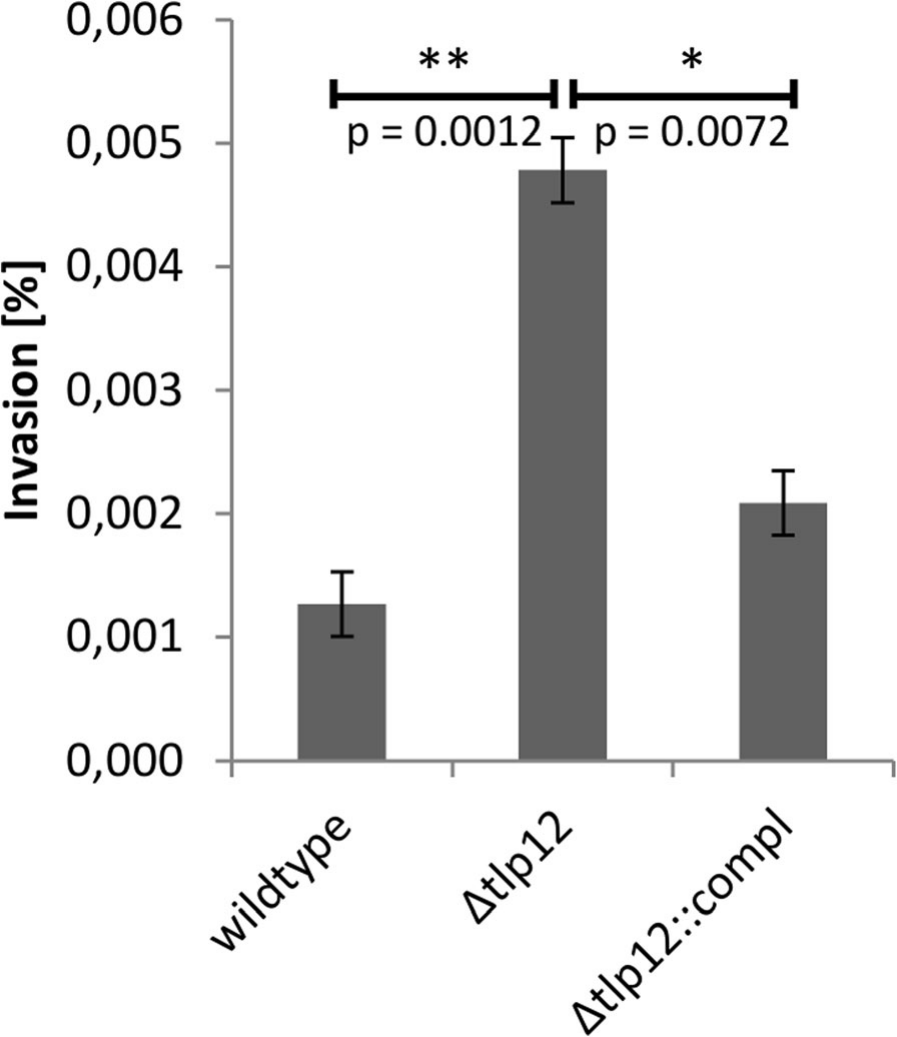
Invasion assay. Invasion rates were determined in Caco-2 cells. Error bars show the standard deviation of a representative experiment (*n* = 3). *p < 0.05; **p < 0.005

**Fig. 5:**
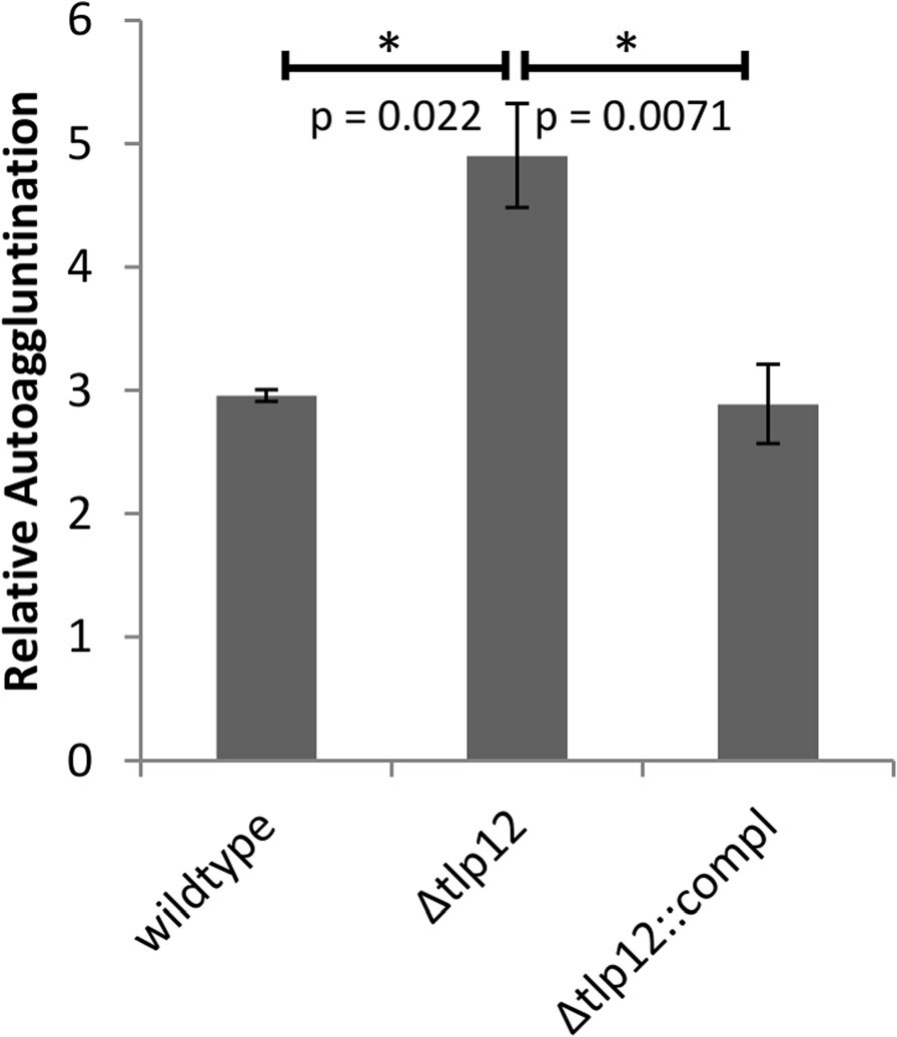
Autoagglutination assay. The experiments were performed in technical duplicates and in biological triplicates. Error bars show the standard deviation of a representative experiment (n = 3). *p < 0.05

**Fig. 6:**
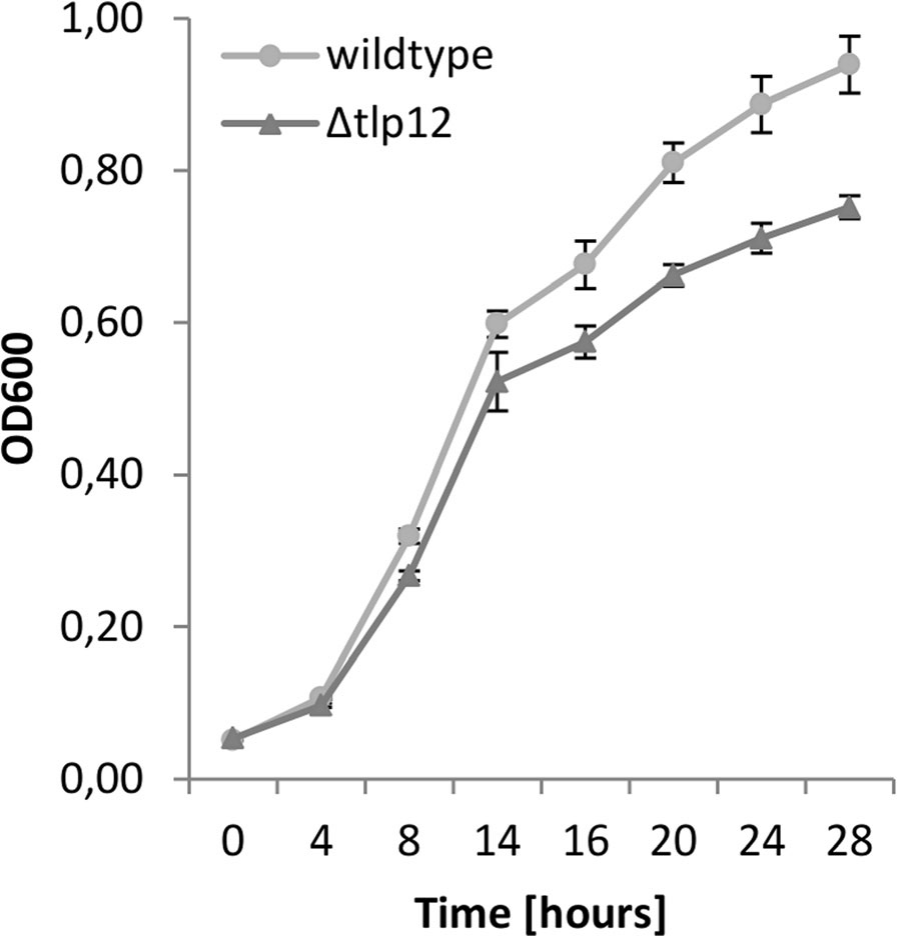
Growth curve. The growth experiments were performed in biological duplicates or triplicates. Error bars show the standard deviation of a representative experiment. *p*-values were calculated for each time point. A significant difference (*p* < 0.05) between wild type and tlp12 mutant was observed for the time points 16 h, 20 h, 24 h and 28 h.

The influence of *tlp12* deletion on the swarming motility of the mutant was measured in a soft agar assay. The diameter of the motility zone produced by the *Δtlp12* mutant was 1.7-fold higher than that of the A17 wild type strain and was reduced almost completely back to wild type level after complementation (Fig. 3). We thus conclude that loss of Tlp12 results in an increased swarming motility in the A17 isolate. The invasion rate of the *Δtlp12* mutant into Caco-2 cells was found to be 4-fold increased and could also be reduced to almost parental strain level after complementation (Fig. 4). In contrast, adhesion to Caco-2 cells was not affected by *tlp12* deletion (data not shown). In addition, the *Δtlp12* mutant displayed unaltered biofilm formation (data not shown). Interestingly the autoagglutination level was 1.7-fold increased in the *Δtlp12* mutant and was restored to almost parental strain level after complementation (Fig. 5). Growth experiments revealed that the duration of the lag phase and the duration of the exponential phase of the *Δtlp12* mutant were unaffected. However, the peak OD values of the *Δtlp12* mutant were significantly reduced compared to the parental strain **(**Fig. 6**)**. This reduced growth rate suggests a direct or indirect effect of Tlp12 expression on *C. jejuni* fitness.

## Discussion

The chemosensitivity of bacteria depends on the repertoire of chemoreceptors expressed by the bacteria, the sensitivity and specificity of the chemoreceptors toward ligands, and on the interplay between different chemoreceptors, which were shown to cooperate in clusters [15]. In this work, the role of the recently identified type A *C. jejuni* chemoreceptor, Tlp12, was studied with the aid of a *tlp12* gene deletion mutant. The *Δtlp12* mutant displayed decreased chemotaxis towards glutamate and pyruvate and was shown to be affected in motility, invasion, growth rate and autoagglutination.

Glutamate has long been known as a chemoattractant for *C. jejuni* [1, 16] and plays a vital role in the *C. jejuni* metabolism. Firstly, glutamate can be transaminated to aspartate by the reaction of glutamate and oxaloacetate to produce aspartate and 2-oxoglutarate. This is catalyzed by the aminotransferase, AspB, in a reversible reaction [17]. 2-Oxoglutarate may then feed into the TCA cycle to produce ATP, while aspartate is deaminated by the aspartase, AspA, into fumarate, which in turn is also fed into the TCA cycle [17]. Secondly, glutamate can react with free ammonia, producing glutamine in an ATP-dependent manner by the action of glutamine synthetase, GlnA [18]. Thus, glutamate provides the link between nitrogen and carbon metabolism and presents the means for nitrogen assimilation in *C. jejuni*. Both genes, *aspB* and *glnA,* are present in the wild type A17 strain (A17*_*00816; A17*_*00757).

Glutamate chemotaxis might be particularly important for the colonization of the chicken gut, since glutamate was shown to be present at very high concentrations in chicken excreta [19, 20]. Chickens are natural hosts for *C. jejuni* and contaminated chicken meat is believed to be a major source for human campylobacteriosis [21]. The predominant colonization site of *C. jejuni* in the chicken gastrointestinal tract is the cecum, where they are present at 10^6^–10^8^ CFU/g of cecal content [22]. *C. jejuni* isolates differ tremendously in their potential to colonize the chicken gut [23, 24] and successful colonization was shown to be a multifactorial process in which a plethora of colonization factors is involved, including chemotaxis genes [25]. Mutants that are deficient in chemotaxis are no longer able to competitively colonize the chicken gastrointestinal tract [2]. Individual chemoreceptors that were demonstrated to have an effect on chicken gut colonization are Tlp1, Tlp4 and Tlp10 [9, 26]. The presence of the Tlp12 glutamate receptor in ~ 30% of the *C. jejuni* isolates might thus be an adaptation to the glutamate- rich environment of the chicken gut and may contribute to colonization of the chicken cecum.

The hypothesis that Tlp12 provides a selective advantage for chicken colonization is strengthened by the fact that the frequency of *tlp12*-positive strains isolated from chicken vs. cattle differs largely. Mund et al. determined the prevalence of the six non-ubiquitous chemoreceptor genes *tlp4*, *tlp5*, *tlp7*, *tlp11*, *tlp12* and *tlp13* in *C. jejuni* isolates of chicken, turkey, bovine and humans [14]. The occurrences of each of the six *tlp* genes in poultry and bovine were normalized for identical sample numbers and expressed as poultry-occurrence-to-bovine-occurrence (poultry/bovine) ratios, in order to identify putative associations of the *tlp* genes with either poultry or cattle (Additional file 3). *Tlp4*, *tlp5*, *tlp7* and *tlp11* were found to be more highly associated with cattle, displaying poultry/bovine ratios of 0.67, 0.67, 0.16 and 0.14, respectively. In contrast, *tlp12* and *tlp13* showed a clear association with poultry, with poultry/bovine ratios of 2.7 and 10.2, respectively.

In addition to glutamate, Tlp12 is also involved in pyruvate recognition. Tlps that respond to pyruvate were identified before and include Tlp6, Tlp8, Tlp9 and Tlp10 [7, 8]. This confirms the general known concept that compound recognition in *C. jejuni* is redundant and that a single ligand can be recognized by more than one chemoreceptor [8, 10], which provides robustness to the *C. jejuni* chemotaxis system.

The Δ*tlp12* mutant was shown to have an increased swarming motility in soft agar. A similar phenotype was described before for two independently generated *C. jejuni Δtlp8* mutants [8, 27] and it was speculated in one of these studies [8], that this phenotype might be due to increased CheY phosphorylation. CheY is the transducer that regulates the flagellar motor. In *E. coli,* phosphorylated CheY reverses the flagellar rotation from run to tumbling and an increased tumbling frequency was shown to lead to a larger swarming zone in soft agar, although a “tumbling only” phenotype has a decreased swarming zone [28]. A CheY null mutant in *C. jejuni* can swim in liquid but fails to develop a swarming zone in soft agar, most likely due to the lack of tumbling [29]. CheY overexpression by a *C. jejuni* diploid *cheY* strain leads to an increased swarming zone [30]. These observations suggest, that as in *E. coli*, the *C. jejuni* phospho-CheY reverses the flagellar rotation from run to tumbling. We thus hypothesize that deletion of *tlp12* might change the interaction of the remaining chemoreceptors with proteins involved in the signal transmission (i.e. CheV, CheA) in a manner that finally results in increased levels of phospho-CheY and thus in a larger swarming zone.

The *Δtlp12* mutant also displayed a higher autoagglutination rate, a phenotype that was previously observed for a mutant of the multi-ligand binding chemoreceptor, Tlp3, in NCTC 11168 [10]. However, in contrast to the *Δtlp3* mutant, no increased biofilm formation ability was detected in the *Δtlp12* mutant.

## Conclusions

The complex and diverging alterations of phenotypic characteristics observed here for Tlp12, but also for previously investigated *tlp* mutants, highlights the complex interaction of the *C. jejuni* chemotaxis system with various other cellular systems. A balanced expression and activity pattern of individual *tlps* appears to be of high importance for adaptive processes in the *C. jejuni* life cycle.

## Methods

### Bacterial culture conditions and cultivation of eukaryotic cells

Bacteria were maintained at 42 °C on Columbia agar plates supplemented with sheep blood (COS) in anaerobe containers with a microaerophilic atmosphere, consisting of 5% O_2_, 10% CO_2_ and 85% N_2_ (CampyGen, ThermoScientific). Human colon carcinoma (Caco2) cells were maintained in 75 cm^2^ cell culture flasks in 30 ml of Dulbecco Minimal Essential Medium (DMEM) supplemented with 10% heat inactivated Fetal Calf Serum (FCS), 1× Non-Essential Amino acids (NEA) and 100 U/ml penicillin and 100 μg/ml streptomycin.

### Growth curves

Bacterial cultures were prepared for growth experiments by firstly growing pre-cultures of the strains in 20 ml Mueller Hinton (MH) medium in 100 ml Erlenmeyer flasks for 24 h at 37 °C and 150 rotations per minute (rpm) under microaerophilic conditions. The ODs were adjusted to OD_600_ 0.05 at the start of the growth experiments by inoculation of 20 ml MH medium with the prepared pre-cultures. Incubation conditions were as described for the pre-cultures.

### Genome sequencing, assembly and annotation

Genome sequencing was carried out on the PacBio *RSII* (Pacific Biosciences, Menlo Park, CA) using P6 chemistry. Genome assembly was performed with the “RS_HGAP_Assembly.3” protocol included in SMRT Portal version 2.3.0. The chromosomal contig was trimmed, circularized, and adjusted to *dnaA* (A17_00001) as first gene. The extrachromosomal element was trimmed and circularized. In addition, genome sequencing of strain A17 was carried out on a NextSeq (Illumina, San Francisco, CA) and quality improvement of the long-read sequence was performed with Burrows-Wheeler Aligner (BWA) [31, 32] mapping the Illumina reads onto the contigs to obtain a final consensus sequence. A final quality of QV60 was confirmed. Automated genome annotation was carried out using Prokka [33]. The complete genome has been deposited at GenBank under accession numbers CP028372 and CP028373.

### Swarming motility assays

Soft agar swarming motility assays were performed as described in [12] with minor modifications. Liquid cultures were grown for 16 h at 37 °C under microaerophilic conditions and then adjusted to an OD_600_ of 0.025. These cultures were stabbed into 0.4% MH agar plates with a 1 μl inoculation loop. The plates were then incubated at 37 °C under microaerophilic conditions for 36 h, after which the diameters of the swarming motility zones were measured.

### Eukaryotic cell invasion and adhesion

Invasion and adhesion assays were performed as described by Everest and coworkers [34] with minor modifications. CaCo2 cells were seeded in 24-well plates at a concentration of 2 × 10^5^/well. After 16 h of incubation, CaCo2 cells were infected with ~ 2 × 10^6^ CFU *C. jejuni* A17 derived mutants, which corresponds to a multiplicity of infection (MOI) of 10. Plates were centrifuged at 600 g for 5 min and incubated afterwards for two hours at 37 °C to allow the bacteria to invade the host cells. An aliquot of the bacterial suspension was plated on COS plates in serial dilutions, for a determination of the actual number of CFU added to each well. Following the 2 h incubation, the bacterial suspension was removed from the CaCo2 cells and the monolayer was washed three times with 1 ml DMEM before the addition of a 1 ml suspension of 100 μg/ml gentamycin in DMEM, followed by an incubation period of two hours at 37 °C. Afterwards, the cell monolayer was washed again three times with 1 ml of DMEM and lysed with 100 μl of 0.1% Triton X-100 in DMEM to release intracellular bacteria. After 10 min of incubation, 900 μl of DMEM was added and the number of viable bacteria in each well was determined by plating serial dilutions on COS plates. Percentage invasion was calculated by dividing the number of invaded bacteria by the number of viable bacteria that were added to the wells. Adhesion experiments were performed in the same way as described above with the only modification being an infection of the cell monolayer for 30 min rather than 2 h and no incubation step with gentamycin. Experiments were performed in technical triplicates and in at least biological duplicates.

### Autoagglutination assay

Autoagglutination assays were carried out as described by Misawa and Blaser [35]. Bacteria grown for 16 h on COS agar plates at 42 °C under microaerophilic conditions were resuspended in PBS (pH 7.4) and adjusted to an OD_600_ of 1. The bacterial suspensions were then added in a volume of 2 ml into glass tubes and incubated for 24 h at 37 °C under microaerophilic conditions without shaking. After incubation, 1 ml of the supernatant was carefully removed and the OD_600_ was measured. Relative autoagglutination was calculated by dividing the initial OD of the bacterial suspension by the final OD of the bacterial supernatant.

### Biofilm assay

The biofilm assays were performed as described by Reeser and coworkers [36] with some modifications. Bacteria grown for 16 h on COS agar plates were resuspended in MH medium and adjusted to an OD_600_ of 0.05. 100 μl of the bacterial suspension was then added to each well of a 96-well plate and the plate was incubated for 48 h at 37 °C under microaerophilic conditions. Only MH medium was added to a separate row of wells to serve as the negative control. Following incubation, the bacterial cultures were removed from each well and the plate was dried for 30 min at 60 °C. 100 μl of 0.1% crystal violet resuspended in water was then added to each well and left to stain for 15 min at room temperature. Unbound crystal violet was removed from the wells, the wells were washed two times with 100 μl of water and the plate was dried for 15 min at 60 °C. The bound crystal violet was decolorized by adding 100 μl solutions of 80% ethanol and 20% acetone into each well for 15 min. After 15 min of decolorization, 90 μl of the bacterial solution was pipetted out of the wells and added to a fresh 96-well plate (as not all of the biofilm might have detached from the wells of the old plate after 15 min). The plate was then read at an absorbance of 570 nm with a microplate reader to quantify the amount of biofilm formed in each well. Results were normalized by subtracting absorbances obtained by the negative controls from these readings. The experiments were done for all strains in technical quadruplets and biological triplicates.

### Δ*tlp12* knockout generation

The *Δtlp12* knock-out mutant was generated by double homologous recombination that resulted in the insertion of a kanamycin resistance cassette, *kanR,* into the gene. For the construction of the knockout vector, a 546 bp 5′-fragment and a 493 bp 3′-fragment of the target gene in the *C. jejuni* strain A24 were amplified with the primers psk-5-TLP12-F/kan-5-TLP12-R and kan-3-TLP12-F/psk-3-TLP12-R, respectively. The psk-5-TLP12-F and psk-3-TLP12-R primers have 30 bp of oligonucleotides that are complementary to the pBluescript SKII vector attached to their 5′ and 3′ ends, respectively. The kan-3-TLP12-F and kan-5-TLP12-R primers have 30 bp of oligonucleotides that are complementary to the ends of the *kanR* gene attached to their 5′ and 3′ ends, respectively. The *kanR* gene was amplified using the Kan 1 and Kan 2 primers. The PCRs were performed as follows: 95 °C for 1 min, 35 cycles at 98 °C for 15 s, 58 °C for 15 s and 72 °C for 1 min and a final incubation at 72 °C for 5 min. The PCRBio HiFi Kit was used for all PCRs and PCR reactions were put together as per manufacturer’s recommendations with 1 U of PCRBIO HIFI polymerase, 400 nM forward and reverse primers and ~ 500 ng of genomic template DNA in 50 μl reactions. The pBluescript SKII vector was digested with BamHI and EcoRI to linearize the vector. The linearized vector, the 3′ and 5′ target gene fragments and the *kanR* gene were assembled using the NEBuilder HiFi DNA Assembly Cloning Kit (NEB) and the assembled knockout vector was transformed into NEB 5-alpha competent *E. coli.* Plasmids were extracted from single colonies and the correct construction of the knockout vector was confirmed by Sanger sequencing using the psk-5-TLP12-F and psk-3-TLP12-R primers. The knockout vector was electroporated into *C. jejuni* A17 wild type competent cells using an ECM600 Electro Cell Manipulator (BTX) with the following settings: resistance – 2.5 kV, capacitance timing – 25 μF and resistance timing – 186 Ω. After electroporation, 100 μl SOC Outgrowth Medium (NEB) was added to the mixture which was then inoculated onto COS plates and incubated for 16 h at 37 °C under microaerophilic conditions to encourage the amplification of the generated mutants. Wild type strains in the background were then excluded by replating the bacteria onto COS plates containing kanamycin (10 μg/ml). Primer sequences are listed in Additional file 4.

### Δ*tlp12* complementation

The complementation of the *Δtlp12* mutation was performed as described in [37] with some modifications. The complementation was performed by a plasmid-based, double-recombinational insertion of the *tlp12*-coding sequence linked to a chloramphenicol resistance cassette (*camR*) into one of the three rRNA loci of the A17 *Δtlp12* mutant. For the construction of the *tlp12* complementation vector, the *tlp12* gene (A17_00255) from the wild type A17 genome was amplified using the TLP12_Complement Primer_Fw and TLP12_Complement Primer_Rev. At its 5′ end, the TLP12_Complement Primer_Fw has a Shine-Dalgarno sequence for improved binding of RNA polymerase after transcription of the gene and 36 bp of an oligonucleotides sequence that is complementary to the linearized pRRC vector at its 5′ end. The TLP12_Complement Primer_Rev primer has 36 bp of oligonucleotides that are complementary to the linearized pRRC vector at its 3′ end. The *tlp12* gene was amplified as follows: 95 °C for 1 min, 35 cycles at 95 °C for 15 s, 65 °C for 15 s and 72 °C for 2 min and a final incubation at 72 °C for 5 min. The PCR reactions were put together as described previously with the PCRBio HiFi Kit. The pRRC vector was digested with *Xba*I and phosphatase treated with Antarctic Phosphatase (NEB), as per manufacturer’s recommendations. The linearized vector and the *tlp12* gene were assembled and transformed into NEB 5-alpha competent *E. coli* as described previously. The correct construction of the knockout vector was confirmed by Sanger sequencing. Electroporation of the complementation vector into A17 *Δtlp12* was performed as described before. The selective chloramphenicol concentration was 12.5 μg/ml. The complementation mutant was named *Δtlp12::*compl. The used primer sequences are listed in Additional file 4.

### Chemotaxis syringe capillary assay

The chemotaxis assays were performed as described by Chandrashehkar et al. [8]. All chemicals tested in the chemotaxis assays were suspended in PBS, adjusted to a pH of 7 and set to a concentration of 100 mM, a concentration that has previously been shown to result in the strongest chemotaxis response [7, 12]. Bacteria grown for 18 h on COS agar plates at 42 °C under microaerophilic conditions were resuspended in 1 ml of PBS (pH 7) and washed once by centrifuging at 4500 g for 5 min. The washed bacterial suspension was then adjusted to an OD_600_ of 0.5. 100 μl of the tested chemical solutions were drawn through a 27 G hypodermic needle (0.40 mm diameter × 20 mm long) into a 1 ml Luer syringe. Buffer alone served as a control. A 100 μl of the adjusted bacterial suspension was then drawn into a 200 μl disposable pipette and the tip was sealed with parafilm. The pipette tip was attached to the needle-syringe system such that most of the needle immersed into the bacterial suspension. The whole system was then incubated horizontally for 1 h at 42 °C under microaerophilic conditions, after which the needle-pipette tip system was detached from the syringe. The bacterial suspension in the syringe was then plated in 10-fold serial dilutions on COS plates for 48 h at 37 °C or 42 °C under microaerophilic conditions and the CFU were counted. The taxis toward a test compound was expressed by calculating the Relative Chemotaxis Ratio (RCR), which is the ratio of the number of bacteria in the syringe with a tested chemical to the number in the control (buffer only) after 1 h incubation. Capillary assays for glutamate and pyruvate were performed at least in biological triplicates, each with at least technical duplicates.

### Statistical analysis

Statistical analysis were performed by two-sided, unpaired Student T-tests using Excel software.

## Additional files

Additional file 1: Amino acid alignment of TLP12of strain A17, TLP12 of strain R14 (H730_01610) and TLP4 of *C. jejuni* NCTC 11168 (cj0262c). (JPG 661 kb)

Additional file 2: Depicts schematics of the double homologous recombination events that resulted in the deletion of *tlp12* and the insertion of the wild type *tlp12* gene into one of the three rRNA clusters in the A17 *Δtlp12* mutant for the complementation of the mutation. (PNG 340 kb)

Additional file 3: Distribution of tlps in poultry and cattle. (DOCX 13 kb)

Additional file 4: Oligonucleotides. (DOCX 18 kb)

## Abbreviations

BLAST: Basic local alignment search tool
CFU: Colony forming units
COS: Columbia agar + sheep blood
DMEM: Dulbecco Modified Eagle Medium
SMRT: Single-molecule real-time
TLP: Transducer-like protein
