# Supplementary figures and images for: The transducer-like protein Tlp12 of Campylobacter jejuni is involved in glutamate and pyruvate chemotaxis
Source: BMC Microbiol. 2018 Sep 10;18:111. doi: 10.1186/s12866-018-1254-0 (PMC6131913; doi:10.1186/s12866-018-1254-0)

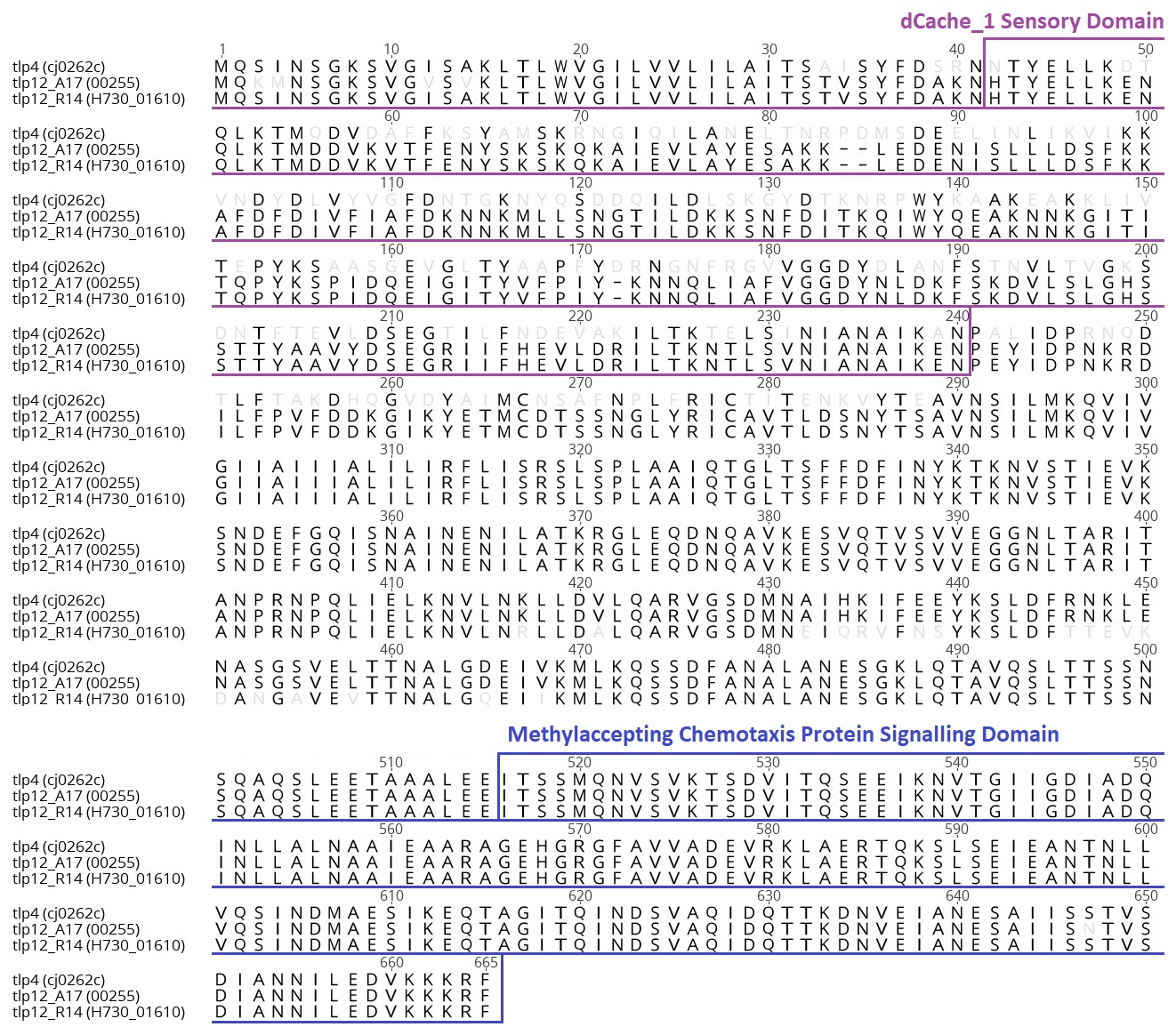

Supplement: Supplementary file 1 — Amino acid alignment of TLP12of strain A17, TLP12 of strain R14 (H730_01610) and TLP4 of C. jejuni NCTC 11168 (cj0262c). (JPG 661 kb) [file 12866_2018_1254_MOESM1_ESM.jpg]

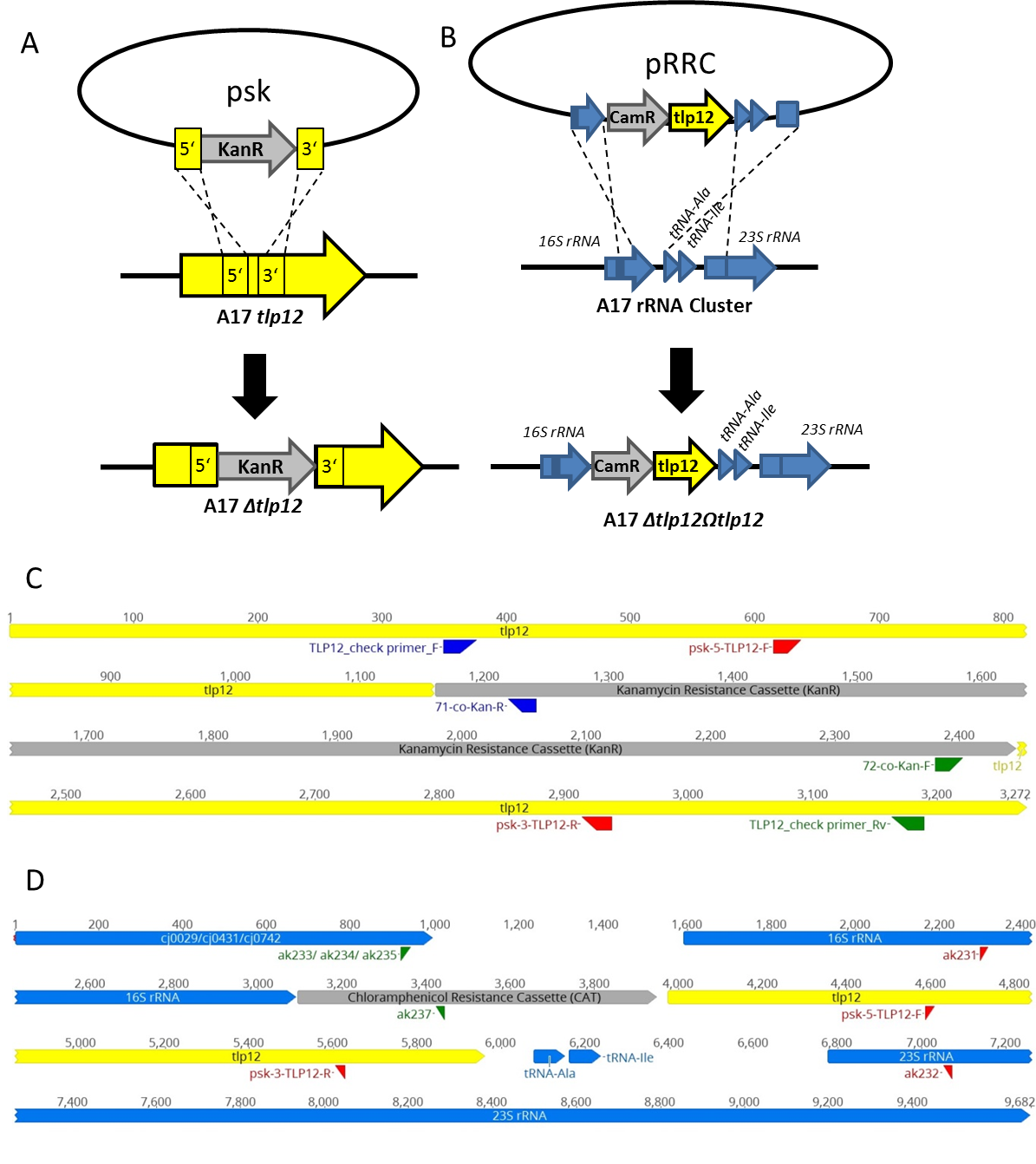

Supplement: Supplementary file 2 — Depicts schematics of the double homologous recombination events that resulted in the deletion of tlp12 and the insertion of the wild type tlp12 gene into one of the three rRNA clusters in the A17 Δtlp12 mutant for the complementation of the mutation. (PNG 340 kb) [file 12866_2018_1254_MOESM2_ESM.png]
