# Supplementary material for: The transducer-like protein Tlp12 of Campylobacter jejuni is involved in glutamate and pyruvate chemotaxis
Source: BMC Microbiol. 2018 Sep 10;18:111. doi: 10.1186/s12866-018-1254-0 (PMC6131913; doi:10.1186/s12866-018-1254-0)
Supplement: Supplementary file 3 — Distribution of tlps in poultry and cattle. (DOCX 13 kb) [file 12866_2018_1254_MOESM3_ESM.docx]

**Additional file 3: distribution of tlps in poultry and cattle**

|  | **Chicken, n=68** | **Turkey, n=24** | **Poultry, n=92** | **Bovine, n=43** | **Bovine, normalized to n=92** | **ratio:Poultry/**  **Bovine** |
| --- | --- | --- | --- | --- | --- | --- |
| tlp4 | 18 | 2 | 20 | 14 | 30 | 0,67 |
| tlp5 | 35 | 9 | 44 | 31 | 66 | 0,67 |
| tlp7 | 4 | 5 | 9 | 27 | 58 | 0,16 |
| tlp11 | 4 | 3 | 7 | 24 | 51 | 0,14 |
| tlp12 | 23 | 7 | 30 | 5 | 11 | 2,73 |
| tlp13 | 32 | 9 | 41 | 2 | 4 | 10,25 |
