# Supplementary material for: The transducer-like protein Tlp12 of Campylobacter jejuni is involved in glutamate and pyruvate chemotaxis
Source: BMC Microbiol. 2018 Sep 10;18:111. doi: 10.1186/s12866-018-1254-0 (PMC6131913; doi:10.1186/s12866-018-1254-0)
Supplement: Supplementary file 4 — Oligonucleotides. (DOCX 18 kb) [file 12866_2018_1254_MOESM4_ESM.docx]

**Additional file 4: oligonucleotides**

| Oligonucleotides | Sequence (5’ to 3’) | Source |
| --- | --- | --- |
| psk-5-TLP12-F | AGGTCGACGGTATCGATAAGCTTGATATCGTGACTCTGAAGGAAGAATTATTT | This Study |
| kan-5-TLP12-R | TCTCGTTTTCATACCTCGGTATAATCTTACACAACTGATACGGTTTGAACTGA | This Study |
| kan-3-TLP12-F | TACTGGATGAATTGTTTTAGTACCTAGATTAGGTGGTAATTTAACAGCAAGAA | This Study |
| psk-3-TLP12-R | GCGGTGGCGGCCGCTCTAGAACTAGTGGATAGCTAAAAGATTGATTTGATCTGCA | This Study |
| Kan 1 (For) | GTAAGATTATACCGAGGTATGAAAACG | (Tareen, et al., 2010) |
| Kan 2 (Rev) | AATCTAGGTACTAAAACAATTCATCCA | (Tareen, et al., 2010) |
| TLP12_check primer_F | TGTCAAATGGAACAATTTTAGATAAAA | This Study |
| TLP12_check primer_R | GCAATTTCAACATTATCTTTAGTAGTT | This Study |
| 71-co-Kan-R | TGGTAGCTTTTTAAATATGGCGC | This Study |
| 72-co-Kan-F | TCAAGCCTGATTGGGAGAAAATA | This Study |
| TLP12 Complement Primer_Fw | GAATTCTGCAGGTACCCGGGATCCACTAGTTCTAGAAGGAGATTTAAATGCAAAAAATGAATTCAG | This Study |
| TLP12 Complement Primer_Rev | GAGACTTATTACTTTGTACTCTAGGGCCGCTCTAGATTAAAACCTTTTCTTCTTAACATCTTC | This Study |
| pRRC-CAT1 | GTGCATCATGCCGTTTGTGAC | (Karlyshev & Wren, 2005) |
| ak231 | CTGGAACTCAACTGACGCTAAG | (Karlyshev & Wren, 2005) |
| ak232 | CTCTTGCACATTGCAGTCCTAC | (Karlyshev & Wren, 2005) |
| ak233 | GCAAGAGTTTTGCTTATGTTAGCAC | (Karlyshev & Wren, 2005) |
| ak234 | GAAATGGGCAGAGTGTATTCTCCG | (Karlyshev & Wren, 2005) |
| ak235 | GTGCGGATAATGTTGTTTCTG | (Karlyshev & Wren, 2005) |
| ak237 | TCCTGAACTCTTCATGTCGATTG | (Karlyshev & Wren, 2005) |
